# Enhanced photodynamic destruction of a transplantable fibrosarcoma using photochemical internalisation of gelonin

**DOI:** 10.1038/sj.bjc.6602600

**Published:** 2005-05-10

**Authors:** A Dietze, Q Peng, P K Selbo, O Kaalhus, C Müller, S Bown, K Berg

**Affiliations:** 1 Department of Radiation Biology, Institute for Cancer Research, The Norwegian Radium Hospital, Montebello, N-0310 Oslo, Norway; 2 Department of Pathology, Institute for Cancer Research, The Norwegian Radium Hospital, Montebello, N-0310 Oslo, Norway; 3 Department of Tumour Biology, Institute for Cancer Research, The Norwegian Radium Hospital, Montebello, N-0310 Oslo, Norway; 4 National Medical Laser Centre, University College London, London, UK

**Keywords:** photochemical internalisation, photodynamic therapy, sarcoma, pharmacokinetics, AlPcS_2a_, gelonin

## Abstract

Photochemical internalisation (PCI) is a technique for releasing biologically active macromolecules from endocytic vesicles by light activation of a photosensitiser localised in the same vesicles of targeted cells. This study investigated the PCI of the toxin gelonin as a way of enhancing the effect of photodynamic therapy (PDT) on a human malignant fibrous histiocytoma transplanted into nude mice using the photosensitiser disulphonated aluminium phthalocyanine (AlPcS_2a_). Pharmacokinetic studies after intraperitoneal administration showed that the serum level of AlPcS_2a_ fitted a biexponential model (half-lives of 1.8 and 26.7 h). The tumour concentration was roughly constant up to 48 h, although fluorescence microscopy showed that the drug location was initially mainly vascular, but became intracellular by 48 h. To compare PDT with PCI, 48 h after intraperitoneal injection of 10 mg kg^−1^ AlPcS_2a_, and 6 h after direct intratumour injection of 50 *μ*g gelonin (PCI) or a similar volume of phosphate-buffered saline (PDT controls), tumour-bearing animals were exposed to red light (150 J cm^−2^). Complete response was observed for more than 100 days in 50% of the PCI tumours but only 10% of the PDT tumours (*P*<0.01). In tumours examined histologically 4 days after light delivery, the depth of necrosis was 3–4 mm after PDT, but 7 mm after PCI. The deeper effect after PCI demonstrates that the light fluence needed to kill tumour is less than with PDT. We conclude that PCI with gelonin can markedly enhance the effect of PDT on this type of tumour and may have a role clinically as an adjunct to surgery to control localised disease.

Photochemical internalisation (PCI) is a new technology, in which photosensitising drugs are used to improve the utilisation of macromolecules in cancer therapy in a site-specific manner (Berg *et al*, 1999). The concept is to localise the sensitiser and the macromolecule in endocytic vesicles of target cells and then excite the photosensitiser with light of an appropriate wavelength (photodynamic therapy, PDT). This releases the endocytically located macromolecules into the cytosol, but only in the irradiated area (PCI). The technique has the advantage of minimal side effects since the effect is localised to the irradiated area. The endosomal escape of macromolecules such as transgene substances can be increased up to a 100-fold or more (Berg *et al*, 1999; Selbo *et al*, 2000). Recently, we have demonstrated a good effect of PCI on two sarcoma cell lines *in vitro* (Dietze *et al*, 2003), although the available *in vivo* data is limited (Selbo *et al*, 2002). The aim of this study was to determine whether PCI could improve the therapeutic effect of PDT on a human soft-tissue sarcoma in a mouse model, particularly to assess whether it might have value as an adjunct to surgical excision of this type of tumour. Soft-tissue sarcomas are highly vascularised tumours with a large interstitial space and thus a low density of tumour cells (Lieberman and Lebovitz, 1990). These properties may influence the pharmacokinetics of the photosensitiser as well as the transport and cellular uptake of the macromolecule of choice.

## MATERIALS AND METHODS

### Chemicals

Disulphonated aluminium phthalocyanine with the sulphonate groups on adjacent phthalate rings (AlPcS_2a_) was provided by Frontier Scientific (Logan, UT, USA). A stock solution of 2 mg ml^−1^ in phosphate-buffered saline (PBS) was kept at −20°C until use. Gelonin was purified from *Gelonium multiflorum* by Dr Gowsala Sivam, Bastyr University, Seattle, USA.

### Tumour model

A human malignant fibrous histiocytoma (MFH) xenograft (TAX1), established in 1988, was provided by Professor Ola Myklebost, Department of Tumour Biology at the Norwegian Radium Hospital, Oslo. It was derived from tissue obtained during resection after local relapse. The tissue was propagated by implantation of fragments (2 mm^3^) in the left hind limb of nude mice (Balb/c nu/nu). These mice were maintained under specific pathogen-free conditions, with food and water supplied *ad libitum*. Housing and all procedures involving animals were performed according to protocols approved by the institutional animal care and use committee, in compliance with the Norwegian Animal Research Authority's guidelines on animal welfare and the guidelines of the UK Co-ordination Committee on Cancer Research (UKCCCR). To minimise the number of animals required, not all combinations of values were studied in the treatment group. Three animals per group were used for the serum and tissue analysis. The tumour size was measured by caliper in two dimensions twice a week and the volume calculated by the formula 0.5 × (length × width^2^). Treatment commenced at a mean volume of 100 mm^3^ (s.d.±25 mm^3^). Animals were killed by cervical dislocation if the volume reached 1000 mm^3^.

### Extraction of the sensitiser from tumour tissue and mouse serum

Animals injected with 10 mg kg^−1^ AlPcS_2a_ i.p. were kept in the dark and 2, 6, 12, 24, 48, 72, 96 and 120 h later, intracardiac blood samples were taken under general anaesthesia terminated by cervical dislocation. After spinning at 5000 r.p.m. for 5 min, the serum was collected and kept frozen until analysed spectroscopically as described below to measure the drug concentration. The entire tumour harvested from the same animal was divided into two parts, one for chemical extraction of AlPcS_2a_ and one for fluorescence microscopy. Both parts were immediately frozen in liquid nitrogen. The tumour tissue used for chemical extraction was thawed, washed twice in PBS, weighed and then digested in 10 ml, 0.1 M NaOH for 4 h at 50°C. After spinning at 3000 r.p.m. for 10 min, AlPcS_2a_ was measured spectroscopically in the supernatant at 672 nm (Perkin-Elmer LS-50B spectroscope with an excitation wavelength of 350 nm and emission detected between 550 and 750 nm). For calibration, measurements were made on tissues extracted from animals not given AlPcS_2a_, but to which known concentrations of AlPcS_2a_ had been added.

### *In vivo* localization of AlPcS_2a_

The frozen tissue blocks for localisation of AlPcS_2a_ were mounted in Tissue Tek II embedding compound (BDH, Poole, UK), and 8 *μ*m sections were cut with a cryostat microtome and mounted on clean glass slides. Fluorescence microscopy was undertaken using a Nikon Eclipse E800 microscope with a 100 W mercury lamp and fluorescence images were made with a highly light-sensitive thermo-electrically cooled charge-coupled device (CCD) camera (Hamamatsu ORCAII-ER, Japan), with a resolution of 1344 × 1024 pixels and a dynamic range of 16 bits per pixel. The equipment used a 330–380 nm excitation filter, a beam splitter and a 590 nm long-pass emission filter. The same lens (× 20) and integrated exposure time (2 s), known to cause less than 5% photobleaching of the dye fluorescence, were used throughout the study. The neighbouring tissue sections were stained with haematoxylin and eosin (H&E) and transmission images made with a colour CCD camera (SPOT-RT, Diagnostic Instruments Inc., MI, USA) to determine the exact microscopic distribution of the dye in the tumour tissues.

For whole body images, four animals were killed 48 h after i.p. injection of AlPcS_2a_ (10 mg kg^−1^) and immediately placed in the light tight chamber of an XFO-6 fluorescence option system equipped with a CCD camera (IVIS™ Imaging System 100; Xenogen Corp. Alameda, CA, USA). The peak AlPcS_2a_ excitation wavelength was 675 nm and emission was measured at 694 nm with a Cy 5.5 filter set, 1 s exposure time and a field of vision (FOV) of 25 cm^2^. Fluorescence emitted from the tumour tissue was used to generate a false colour image using LivingImage Software (Xenogen Corp.).

### Western blotting

The abundance and stability of gelonin in the tumour tissue after intratumour (i.t.) injection was investigated by chemical extraction and Western blotting. Animals received a single i.t. injection of 50 *μ*g (25 mg kg^−1^ b.w.) gelonin dissolved in PBS (pH 7.2) and were killed 10 min, 1, 6, 24 or 48 h later. Control animals received no gelonin. At post mortem, the tumours were excised, snap frozen in liquid nitrogen and freeze-fractured with a Bio-Pulverizer (BioSpec Products, Bartlesville, OK, USA) cooled thoroughly with liquid nitrogen. Then, tissue lysate was prepared by homogenisation in a modified RIPA buffer (150 mM sodium chloride, 50 mM Tris-HCl, pH 7.4, 1 mM EDTA, 1 mM phenylmethylsulphonyl fluoride, 1% NP-40, 0.25% Na-deoxycholate, 1 mM Na_3_VO_4_, 1 mM Na-fluoride and 10 *μ*l ml^−1^ protease inhibitor cocktail (Sigma, Louis, MO, USA)) for 1 h at 4°C. Subsequently, tissue and cell debris were removed by centrifugation and the protein concentration of the supernatant was determined with a modified Lowry assay (Bio-Rad, Hercules, CA, USA). The tissue lysates were boiled for 5 min in 1 × SDS sample buffer (50 mM Tris-HCl pH 6.8, 12.5% glycerol, 1% sodium dodecylsulphate, 0.01% bromophenol blue), separated by SDS–PAGE (100 *μ*g protein well^−1^) followed by overnight transfer to a PVDF membrane (Amersham Biosciences, Buckinghamshire, England). The membrane was then blocked in a TTBS buffer containing 5% dry-milk and afterwards probed for 1 h at room temperature (RT) with a rabbit polyclonal antibody (SIFF, Oslo, Norway) against gelonin. The membrane was subsequently washed 3 × 10 min (RT) with TTBS buffer followed by probing with a HRP-linked goat-anti-rabbit antibody in TTBS buffer containing 5% dry-milk for 1 h at RT. To visualise the specific bands of gelonin, the ECL Plus™ (Amersham) Western blotting detection kit was used. Chemifluorescence was detected by the Storm® gel and blot imaging system (Amersham).

### Photodynamic therapy and PCI

Tumour-bearing mice were allocated randomly to one of five groups: 1: PCI, 2: PDT, 3: no treatment, 4: AlPcS_2a_+gelonin (no light) and 5: gelonin+light (no AlPcS_2a_). Groups 1, 2 and 4 were injected with 10 mg kg^−1^ i.p. AlPcS_2a_ and kept in the dark for 48 h prior to light delivery (no light in group 4). Injection of 25 *μ*l of gelonin (2 mg kg^−1^, groups 1, 4 and 5) or PBS (groups 2 and 3) directly into the centre of the tumour was performed 6 h prior to delivery of red light from a 150 W halogen lamp (Xenophot, HLX64640) filtered through a 580 nm long-pass and a 700 nm short-pass filter (150 mW cm^−2^, total dose 150 J cm^−2^). For light delivery, the mice were fixed in a holder and the area surrounding the tumour was completely covered with aluminium foil. The mice were then kept in subdued light for a further 6 days. Tumour size was documented twice a week. If there was apparent eradication, monitoring was reduced to once a week to look for any tumour recurrence. For histological assessment, separate animals were killed 4 days after light delivery and the tumours divided at the centre of the zone of necrosis into two parts in the plane parallel to the axis of light delivery. The specimens were fixed in 4% formalin for subsequent sectioning and staining with H&E. All histological sections were examined microscopically to document the extent of viable tumour tissue. The first cuts, representing the largest cross-section in the zone of necrosis were imaged by a scanner and hard copies printed for further estimation of the areas of viable and nonviable tumour.

### Analysis and statistics

The AlPcS_2a_ serum concentration kinetics was analysed by nonlinear regression of log concentration *vs* the log multiexponential decay with time. Significant differences between time points were assessed by one-way analysis of variance. Significant differences between PDT and PCI treatment and the histological findings after the treatment were assessed by *t*-test analysis.

## RESULTS

### Serum and tumour pharmacokinetics

**Figure 1:**
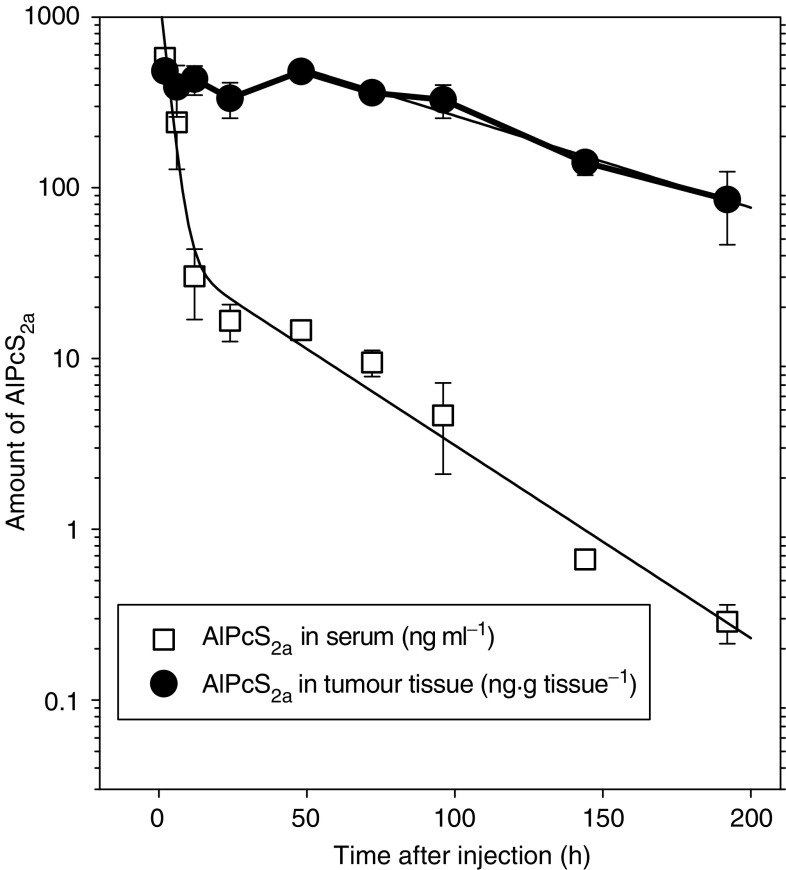
Time-dependent concentration of AlPcS_2a_ in serum and tumour after i.p. injection of 10 mg kg^−1^. Regression lines are used to describe the decay of AlPcS_2a_ levels from 48 h after administration. Plots are the mean values of 3–5 animals for each time point. Bars, s.d.

Figure 1 shows the variation with time of the AlPcS_2a_ level in the mouse serum and tumour after i.p. administration. At 2 h after administration, the serum level was relatively high (∼600 ng ml^−1^), but this declined rapidly. The serum clearance did not follow a simple monoexponential decay, but fitted well to a biexponential two-compartment model described by the equation *C*=1420e^−0.39*t*^+41.8e^−0.026*t*^ (corr. coeff. *R*=0.9916). The model gives two half-lives of 1.8±0.58 and 26.7±2.9 h, correlating to elimination rate constants of 0.39±0.13 and 0.026±0.003 h^−1^. A three-compartment model did not improve the correlation with the observations.

One-way analysis indicated that the amount of AlPcS_2a_ in the tumours was not significantly different between 2 and 48 h after administration (*P*=0.62) and was about 400ng g^−1^. Beyond 48 h, AlPcS_2a_ was cleared from the tumour with an apparent elimination rate constant of 0.0124 h^−1^ (*C*=914e^−0.0124*t*^). This constant is based on the AlPcS_2a_ concentration in the tumour (ng g^−1^ tissue) and is thus biased by the simultaneous growth of the tumour. The tumour doubling time in these experiments was 147±6 h following the regression line *V*=85.5e^0.047*t*^ mm^3^ (corr. coeff. *R*=0.997). The corrected elimination decay may thus be described as *A*=[914e^−0.0124*t*^] × [0.0855e^0.0047*t*^]=78e^−0.0077*t*^, with the approximation that 1 g tissue equals 1 ml. The corrected elimination rate constant is 0.0077±0.0010 h^−1^, which indicates that the tumour growth compensates for only 0.0047/0.0124=38% of the tumour concentration decay.

### *In vivo* localisation of AlPcS_2a_ in tumour-bearing mice

**Figure 2:**
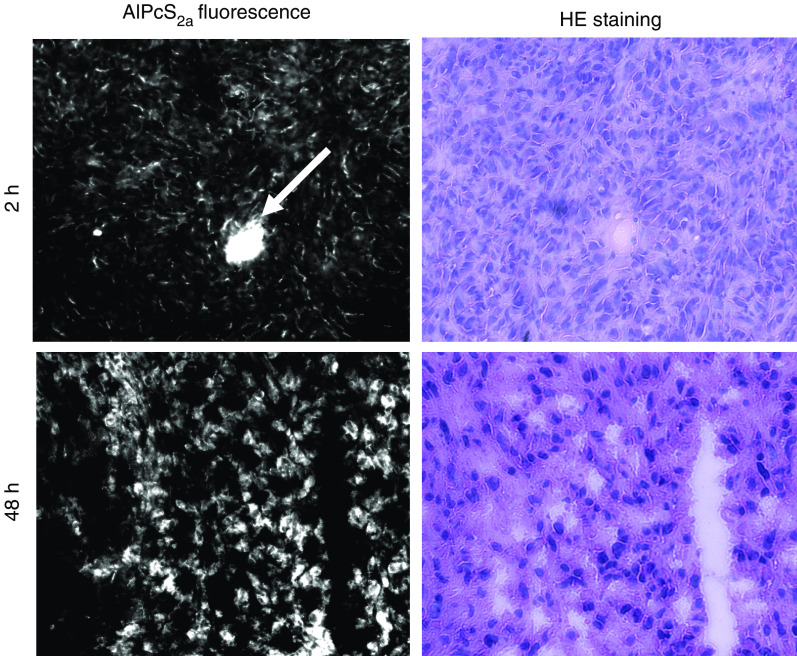
Distribution of AlPcS_2a_ in tumours after i.p. injection of 10 mg kg^−1^ AlPcS_2a_. Upper photos (fluorescence and H&E staining): 6 h after injection, AlPcS_2a_ is mainly in the tumour vasculature. Lower photos (fluorescence and H&E staining): 48 h after injection, AlPcS_2a_ is mainly intracellular.

**Figure 3:**
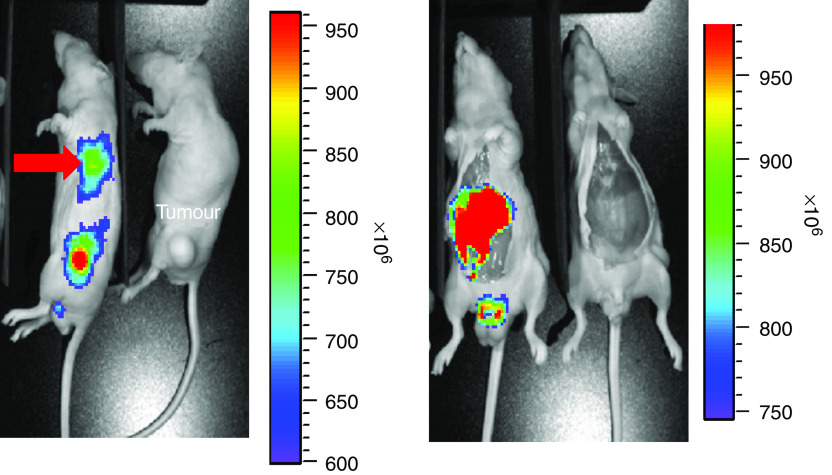
Macroscopic fluorescence 48 h after i.p. injection of 10 mg kg^−1^ AlPcS_2a_. High fluorescence intensity is red and low intensity is blue. High intensity was detected in the tumour region and in the liver.

A major requirement for PDT and PCI is that the photosensitiser should localise into the area to be treated. In addition, PCI requires the photosensitiser to be located in the same endocytic vesicles of parenchymal cells as the macromolecule of interest. Localisation in the blood or the tissue stroma will reduce the synergism. Our pharmacokinetic analysis indicated that AlPcS_2a_ was essentially eliminated from the serum within 24 h, while the concentration in tumour tissue was stable from 2 to 48 h after administration. Thus, the *in vivo* localisation of AlPcS_2a_ was evaluated 2 and 48 h after administration by fluorescence microscopy. The cryosections obtained 2 h after sensitiser injection (Figure 2A) showed a fluorescence pattern indicating AlPcS_2a_ to be mainly in and near vascular structures as well as in the stromal compartment of the tumour, but with none in individual tumour cells. In contrast, sections obtained after 48 h (Figure 2B) showed a more diffuse fluorescence concentrating in the tumour cells. Although limitations in the resolution of cryosections do not allow precise determination of intracellular localisation, the nuclei seem low in AlPcS_2a_. Photosensitisers as used in PDT generally tend to accumulate in neoplastic lesions (Dougherty *et al*, 1998). To verify directly the accumulation of AlPcS_2a_ in tumour tissue, whole-body fluorescence imaging was used. Fluorescence emitted from tumour tissue was detected 48 h after photosensitiser injection (Figure 3A). Dissection of the animal verified that the low signal detected intraabdominally was from the liver (Figure 3B). The distribution pattern of AlPcS_2a_ fluorescence was similar after intravenous injection (data not shown).

### Quantification of gelonin distribution after intratumoral injection

**Figure 4:**
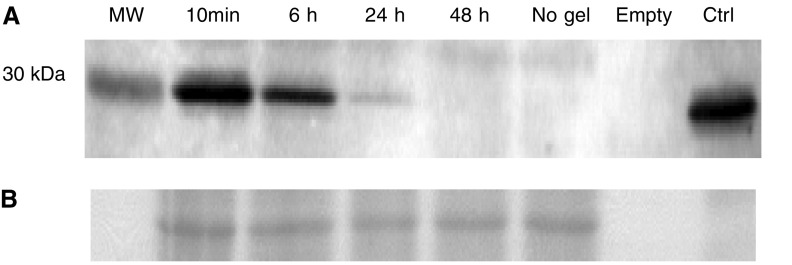
(**A**) Western blot showing level of gelonin in tumour at times from 10 min to 48 h after direct i.t. injection of 50 *μ*g. The blot shows also an MW standard of 30 kDa as well as pure native gelonin as control (ctrl) and tumour extract from untreated tissue (no Gel). (**B**) The PVDF membrane was stained with Ponceu S solution to verify even loading.

A prerequisite for photochemical activation of gelonin is its stable presence in the tumour cells after intratumor injection. Gelonin may be excreted by the highly vascularised sarcomas or degraded by hydrolases. Figure 4 shows that undegraded gelonin is still in the tumour 6 h after injection, although reduced from the amount injected. Traces of gelonin can be seen even 24 h after injection. It cannot be determined from these results whether the attenuation of the gelonin content in the tumour tissue is due to elimination into the blood or intratumoral degradation. No traces of degradation products were observed on the Western blots.

### PDT and PCI effect on tumour growth

**Figure 5:**
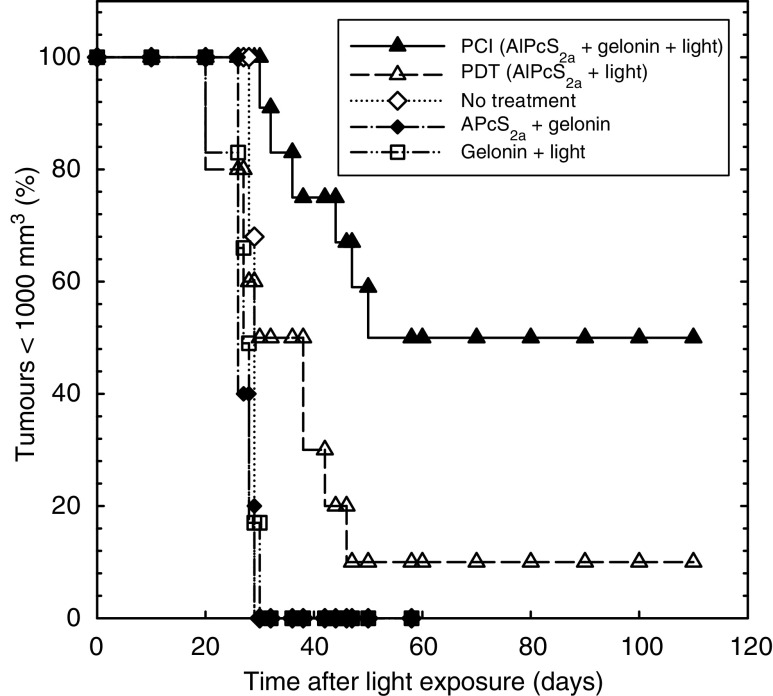
Tumour growth after treatment (Kaplan–Meier plot). The five groups treated were: group 1: PCI, group 2: PDT, group 3: no treatment, group 4: AlPcS_2a_+gelonin (no light), group 5: gelonin+light (no AlPcS_2a_). Animals were killed when the tumour volume reached 1000 mm^3^.

The time for tumours to reach 1000 mm^3^ (our chosen end point, at which time animals were killed by cervical dislocation) was the same (31±6 days) in all control groups (no treatment, gelonin and light, AlPcS_2a_ with gelonin in the absence of light (Figure 5, data not shown)). Of 10 tumours treated with PDT, only one showed a complete response and four showed a growth delay of approximately 17 days. The growth curves of the others were indistinguishable from the controls. In animals treated with PCI, most tumours disappeared by 15 days after the treatment. In total, 50% of these animals (six of 12) remained tumour free although in the other 50%, the tumours started to grow again by 20 days, taking 43±9 days to reach 1000 mm^3^ (a delay of up to 30 days compared with the controls (*P*<0.01) and up to 11 days compared with PDT-treated animals (*P*<0.01)). There were no significant differences in tumour volume at the time of light delivery between the complete remission group and those that regrew later. All tumour-free animals were followed for 110 days.

**Figure 6:**
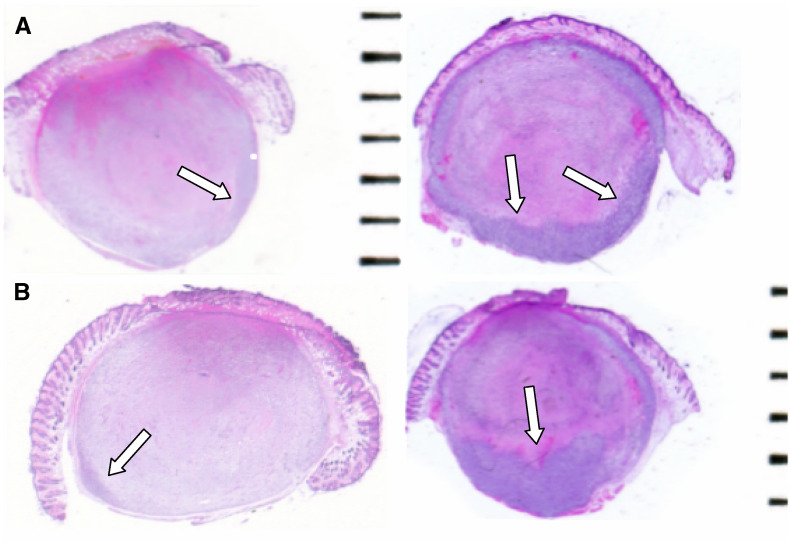
Photomicrographs of tumours 4 days after treatment. Areas of complete necrosis were clearly demarcated from adjacent viable tissue (arrows). (**A**) Tumour treated with PCI. (**B**) tumour treated with PDT.

Histological examination of the central area of tumours 4 days after treatment showed that in the PDT group, a mean of 25% of the tumour was viable compared with 2.5% in the PCI group (*P*<0.001) (Figure 6). Measured from the surface of the skin, the PDT effect was typically 3–4 mm deep compared with 6–7 mm (and usually reaching the bottom of the tumour mass) for the PCI group (Figure 6). Intratumour injection of gelonin or PBS with or without light application induced a small amount of necrosis in the centre of the tumour, presumably due to a local pressure effect. However, this had no influence on the survival curve (Figure 5) or the tumour growth (data not shown).

## DISCUSSION

Photodynamic therapy is a versatile treatment modality for cancer and is approved for use in the treatment of a variety of malignant and premalignant conditions (Dougherty, 2002; Dolmans *et al*, 2003). However, one of its main limitations is the relatively poor penetration of red light into tissue due to the absorption by natural pigments such as haemoglobin and melanin. Photodynamic therapy is therefore best suited to small and thin lesions (Dougherty *et al*, 1998; Moan *et al*, 1998). However, PCI of gelonin has been shown to reduce the fluence needed to obtain the same cytotoxic effect as with PDT (Selbo *et al*, 2001; Dietze *et al*, 2003), so increasing the depth of effect that can be achieved in solid tumours. It was therefore of particular interest to study the potential of PCI for the treatment of a solid tumour like the sarcoma studied here. The structural properties of soft-tissue sarcomas may influence the pharmacokinetics of the photosensitiser as well as the possibility to activate the therapeutic potential of macromolecules such as gelonin photochemically.

The serum pharmacokinetics of AlPcS_2a_ in this study best fitted a biexponential decay. The initial rapid elimination phase, representing the distribution of the photosensitiser to the various organs was followed by a second phase representing drug elimination. The two half-lives are similar to those for the clearance of m-THPC (Cramers *et al*, 2003; Jones *et al*, 2003). At the start of the second phase (about 24 h after administration), the serum concentration is approximately 30-fold lower than in tumour tissue, so from this time onwards, there is unlikely to be a significant PDT effect on blood components under the conditions required for tumour necrosis.

The tumour concentration of AlPcS_2a_ also showed a two-phased kinetic pattern, but with a stable concentration of about 400 ng g^−1^ tissue during the first 48 h after administration followed by a single exponential decay. *In vitro* studies indicate that photosensitisers entering cells by endocytosis tend to be retained in these vesicles for a long time (>30 h) as long as they are not released into the cytosol by light activation (unpublished data). The tumour clearance rate for AlPcS_2a_, corrected for tumour growth indicates a half-life of 90 h (56 h uncorrected). This stable plateau of photosensitiser concentration in tumour tissue for 1–2 days after administration followed by clearance over several days has been reported for several photosensitisers (Peng *et al*, 1991b). The half-life for AlPcS_2a_ in the tumour tissue is similar to or shorter than that of mTHPC and does not indicate that AlPcS_2a_ is retained in the tumour tissue for a longer time than other photosensitisers despite its expected retention in endocytic vesicles.

At 48 h after administration, the whole-body fluorescence images clearly showed a greater retention of AlPcS_2a_ in the tumour than in the surrounding tissue and the fluorescence microscopy showed that the drug is localised in tumour cells. This would appear to be the best time interval between drug administration and light delivery. These results correlate well with other reports and indicate that the structural properties of the sarcoma tissue do not spoil the possibility of using PCI as a new treatment modality (Peng and Moan, 1995; Selbo *et al*, 2001). The results are also in accordance with previous studies, which indicated that AlPcS_2a_ is located in the cells of an experimental malignant melanoma rather than in the interstitium (Peng *et al*, 1991a, 1991b), and that the compound has a high tumour to tissue ratio (Chan *et al*, 1990).

Our results showed an increased survival time in tumour-bearing animals using the combination of the photoactivation of AlPcS_2a_ and gelonin (PCI) compared with PDT alone (light and AlPcS_2a_) or gelonin alone. The 50% complete response rate observed after PCI correlated with necrosis reaching a depth of 7 mm and covering a mean of more than 97% of the cut surface of tumours examined 4 days after light delivery. After PDT, the depth of necrosis was no more than 3–4 mm. This may indicate that tumour eradication is related to a threshold number of tumour cells surviving the treatment, and that this threshold may be around 2.5%. In this study, the gelonin was given by direct i.t. injection, so it is difficult to know exactly where it was located, but it may also be possible to administer it systemically, when one could be more confident that it reached all the cells in the target area. Our findings also provide evidence that PCI using gelonin effectively reduces the light fluence needed to obtain a cytotoxic effect inside tumours compared with PDT alone (Berg *et al*, 1999; Selbo *et al*, 2001). The fluence rate of 670 nm light is reduced by 50% at a depth of 3 mm (Moan *et al*, 1998).

This study was undertaken to investigate the possibility of using PCI in the treatment of sarcoma patients before, during or after surgery, particularly to reduce the incidence of local recurrence. At present, 50% of patients die within 5 years due to local relapse or metastases (Dirix and Van Oosterom, 1999). Chemotherapy and radiation therapy often do not give satisfactory results and have the disadvantages of troublesome side effects. Photochemical internalisation has few side effects since the treatment effect is localised to the illuminated area apart from skin photosensitivity due to the photosensistiser, which is reasonably easy to control. New developments in molecular biology have led to experimental therapeutic approaches designed specifically to target changes in malignant cells (Hunt and Feig, 1999). Previously we reported the possibility of using PCI for the targeted delivery of macromolecules to cancer cells (Selbo *et al*, 2000; Prasmickaite *et al*, 2002; Kloeckner *et al*, 2004). The combination of light-directed therapy and molecular targeting which is possible using PCI could become a powerful adjuvant treatment modality, which could meet the needs of many patients with localised tumours that are difficult to eradicate with current techniques.
